# Hematological and iron status in aerobic vs. anaerobic female athletes: an observational study

**DOI:** 10.3389/fspor.2024.1453254

**Published:** 2024-11-11

**Authors:** Doaa A. Osman, Mohamed Ismail Elassal, Hamada Ahmed Hamada, Reham Hamed Saad Hamza, Hoda Mohammed Zakaria, Reem Alwhaibi, Gehan A. Abdelsamea

**Affiliations:** ^1^ Department of Physical Therapy for Women’s Health, Faculty of Physical Therapy, Cairo University, Giza, Egypt; ^2^ Department of Physical Therapy for Pediatrics, Faculty of Physical Therapy, Cairo University, Giza, Egypt; ^3^ Department of Physical Therapy, Faculty of Applied Medical Sciences, Al Zaytoonah University, Amman, Jordan; ^4^ Department of Biomechanics, Faculty of Physical Therapy, Cairo University, Giza, Egypt; ^5^ Department of Physical Therapy for Women’s Health, Faculty of Physical Therapy, Heliopolis University for Sustainable Development, Cairo, Egypt; ^6^ Department of Physical Therapy for Neurology, Faculty of Physical Therapy, Cairo University, Giza, Egypt; ^7^ Department of Rehabilitation Sciences, College of Health and Rehabilitation Sciences, Princess Nourah Bint Abdulrahman University, Riyadh, Saudi Arabia; ^8^ Department of Physical Therapy for Women’s Health, Faculty of Physical Therapy, Delta University for Science and Technology, Gamasa, Egypt

**Keywords:** hematological factors, transferrin, ferritin, aerobic training, anaerobic training, athletic females, adolescence

## Abstract

**Introduction:**

Physical training induces iron status impairment in athletic females in the short term and over prolonged periods. Nevertheless, the existing literature lacks a comprehensive evaluation of the differential impacts of aerobic vs. anaerobic training on hematological indices and iron status among adolescent female athletes. The aim of this study was to assess the hematological factors and iron status in aerobic vs. anaerobic training in athletic females.

**Methods:**

This observational, cross-sectional study recruited twenty-five adolescent athletic females; thirteen of them participated in an aerobic sport (long-distance running), while twelve of them participated in an anaerobic sport (broad jumping). Hematological factors were assessed by analyzing blood concentrations of hemoglobin (Hb), hematocrit (Hct), red blood cell (RBC) count, mean corpuscular volume (MCV), and mean corpuscular hemoglobin concentration (MCHC), while the iron status assessment was conducted through evaluating levels of serum transferrin and serum ferritin.

**Results:**

Athletic females who participated in the aerobic sport showed significantly lower Hb (MD −0.84; 95% CI −1.63: −0.04; *p* = 0.041), Hct (MD −5.49; 95% CI −7.86: −3.12; *p* = 0.0001), RBC count (MD −0.37; 95% CI −0.57: −0.17; *p* = 0.001), and MCV (MD −5.15; 95% CI −9.41: −0.89; *p* = 0.020), as well as significantly higher MCHC (MD 2.99; 95% CI 2.18: 3.79; *p* = 0.0001) and serum transferrin (MD 46.77; 95% CI 10.95: 82.59; *p* = 0.013) than athletic females who participated in the anaerobic sport. However, there was an insignificant difference in serum ferritin levels (MD −3.18; 95% CI −11.49: 5.13; *p* = 0.437) between both groups.

**Conclusion:**

Except for the ferritin level that exhibited an insignificant difference between aerobic and anaerobic training, aerobic training was associated with a worse impact on the hematological factors and iron status than anaerobic training in adolescent athletic females.

## 1 Introduction

Athletes, especially adolescent menstruating athletic females, have a high risk of anemic and non-anemic iron deficiency because of negative iron balance (high iron requirements and inadequate nutritional intake), excessive loss of iron (sweating, menstruation, gastrointestinal bleeding, and exercise-induced hemolysis), and exercise-induced acute inflammation (an inflammatory response that occurs after training) (1, 2). The complexity of iron metabolism in athletes further exacerbates this risk. Recent research has highlighted the pivotal role of hepcidin, a peptide hormone, in regulating iron balance. Post-exercise inflammatory responses trigger an upregulation of inflammatory markers, particularly interleukin-6, which in turn stimulates hepcidin production (3–5). This hepcidin response can significantly impair iron absorption and recycling, potentially aggravating iron deficiency in athletes (6, 7).

Furthermore, studies found that different types of exercises with varying intensities and durations, whether performed in a single session or over a lengthy period, have a major effect on components of blood (cellular content and plasma volume) in athletic, non-athletic, healthy, and unhealthy individuals (8–13). These exercise-induced changes in blood components can further complicate the assessment and management of iron status in athletes. Additionally, recent studies have explored the potential impact of low energy availability and low-carbohydrate or ketogenic diets on hepcidin response and iron metabolism in athletes, suggesting that dietary factors may play a more significant role in iron status than previously thought (14, 15).

Iron is a key micronutrient for hemoglobin (Hb), myoglobin, and enzyme formation. It is necessary for the transport of oxygen, production of energy, and performance of sports since it prevents muscle fatigue, raises maximum oxygen consumption, and lowers blood lactate levels (16, 17). Iron's role extends beyond these well-known functions, as it is also crucial for the electron transport chain, DNA synthesis, and overall energy metabolism. The importance of iron in biological processes is underscored by its ability to facilitate redox reactions, acting as a cofactor for various enzymes and proteins (1, 18). However, it's worth noting that iron can also be toxic at high levels due to its potential to contribute to the formation of reactive oxygen species, highlighting the need for careful regulation of iron status in athletes (19).

Therefore, the compromised iron status in athletic females negatively impacts their sport performance (20). This impact can manifest in various ways, affecting not only endurance capacity but also strength, immune function, fatigue levels, and mood status (21). Iron deficiency without anemia can impair metabolic systems with iron-containing proteins, potentially reducing oxidative capacity and the muscles’ ability to use oxygen efficiently (1, 22, 23). When iron deficiency progresses to anemia, the reduced oxygen-carrying capability of the blood can lead to a significant decrease in maximal oxygen consumption and endurance capacity (24, 25).

Sports can be categorized according to the type of training into anaerobic or aerobic. Anaerobic sports involve activities of short duration and high intensity, while aerobic sports involve activities of longer duration and lower intensity (26). Although previous research has studied the effect of aerobic vs. anaerobic athletic training on iron-related parameters (27, 28), there is a significant gap in understanding these effects, specifically in adolescent athletic females. This population is of particular interest due to its unique physiological state, combining the demands of growth and development with the stresses of athletic training (29).

Consequently, this study aimed to assess the hematological factors and iron status in aerobic vs. anaerobic training in adolescent athletic females. This study might expand the role of physiotherapy in the field of women's health and sports medicine during adolescence. It could provide valuable information for developing targeted interventions to prevent and manage iron deficiency in adolescent female athletes, considering the specific demands of aerobic and anaerobic sports. By addressing these issues, this research has the potential to contribute to the overall health and performance optimization of adolescent female athletes.

## 2 Materials and methods

### 2.1 Design

The study was an observational cross-sectional design. Ethical approval was provided by the institutional review board of the Faculty of Physical Therapy, Cairo University [No: P.T.REC/012/004727] and ClinicalTrials.gov ID NCT05707455. The data collection phase spanned the months of September and October 2022, in compliance with the ethical guidelines set forth by the Declaration of Helsinki, which governs research involving human participants.

### 2.2 Recruitment

A sample of twenty-five adolescent athletic females was enrolled in this study; thirteen of them were female long-distance runners, while the other twelve were female broad jumpers. They were selected through advertisements from local track and field clubs in Cairo, Egypt, which specialize in both aerobic and anaerobic athletic disciplines. Subsequent to the clarification of the study's nature, aim, and benefits, written informed consent was signed by the parents or legal guardians of the adolescent females, emphasizing the privacy of their information and their ability to decline participation or withdraw consent at any point.

### 2.3 Eligibility criteria

To be involved in this study, the participants’ age was 16–19 years old, and they had a body mass index (BMI) <25 kg/m^2^. This age range was chosen to focus on adolescent female athletes, a critical period for iron status development (30), and an understudied population in sports medicine research. To minimize the impact of hormonal variability during adolescence, all participants were required to have regular menstrual cycles of 21–35 days (31) for at least the past 6 months, confirmed by a self-administered questionnaire. They were healthy, athletic, virginal, and non-smokers; they followed a normal, balanced diet, including all food groups; had the same socioeconomic status; had a weekly training workload of 10–20 h with an average of 14 h per week; had training experience of 5–9 years; and didn't use any medications or hormonal treatment that could affect iron status. The participants were competitive athletic females engaged in regular, structured training at local track and field clubs, but not at a professional level. The aerobic group consisted of female long-distance runners, while the anaerobic group consisted of female broad jumpers. Their training regimens varied accordingly: the aerobic group focused on endurance runs, supplemented by interval and strength training, whereas the anaerobic group concentrated on plyometric and jump exercises, along with sprint, acceleration, and strength training. The participants were excluded if they followed a vegetarian diet, or had amenorrhea, heavy menstrual bleeding (menstrual flow lasting more than 7 days with changing pads every 1–2 h) (32), current infection, chronic inflammation, diabetes, hypertension, cardiovascular diseases, hematological diseases (except iron deficiency with or without anemia), or recent blood transfusion either donor or recipient (33, 34).

### 2.4 Outcome measures

#### 2.4.1 Anthropometric measures

The weight, as well as height, were evaluated for each athletic female in the two groups by a weight-height scale. Then, the weight was divided by the height squared (Kg/m^2^) to calculate the BMI.

#### 2.4.2 Training profile

The training profile of each athletic female in both groups was assessed through a self-administered questionnaire, including questions about the age of participation in sport (years) and weekly training workload (hours). Then, the athlete's age of participation in sport was subtracted from her chronological age to calculate the training experience (years).

#### 2.4.3 Hematological factors and iron status parameters

After an overnight fast and a period of rest, blood sampling was conducted in the morning timeframe of 8–9 AM. Blood samples were collected during the early follicular phase (2nd or 3rd day) of the menstrual cycle to standardize hormonal conditions. The athletic females were requested to avoid exercise performance on the preceding day to sampling. Blood was drawn from the antecubital vein into one ethylene diamine tetraacetic acid (EDTA) tube and one beads tube for serum separation (S-Monovette KE, Germany). In the EDTA samples, Hb, hematocrit (Hct), red blood cell (RBC) count, mean corpuscular volume (MCV), and mean corpuscular hemoglobin concentration (MCHC) were examined via an automated cell counter (Sysmex XS 1000, Japan). The serum sample was centrifuged, and the following parameters were calculated: serum transferrin (COBAS Integra 400 Plus, Switzerland) and serum ferritin [ADVIA Centaur XPT (Siemens), Germany]. All athletic females’ samples were run in the same assay to decrease any variance from inter-assay variability.

Participants were characterized as having normal iron status (Hb levels above 12 g/dl alongside serum ferritin levels above 16 ng/ml), iron deficiency without anemia (Hb levels above 12 g/dl alongside serum ferritin levels below 16 ng/ml), or iron deficiency with anemia (Hb levels below 12 g/dl alongside serum ferritin levels below 16 ng/ml) (35).

### 2.5 Sample size estimation and statistical analysis

**Figure 1:**
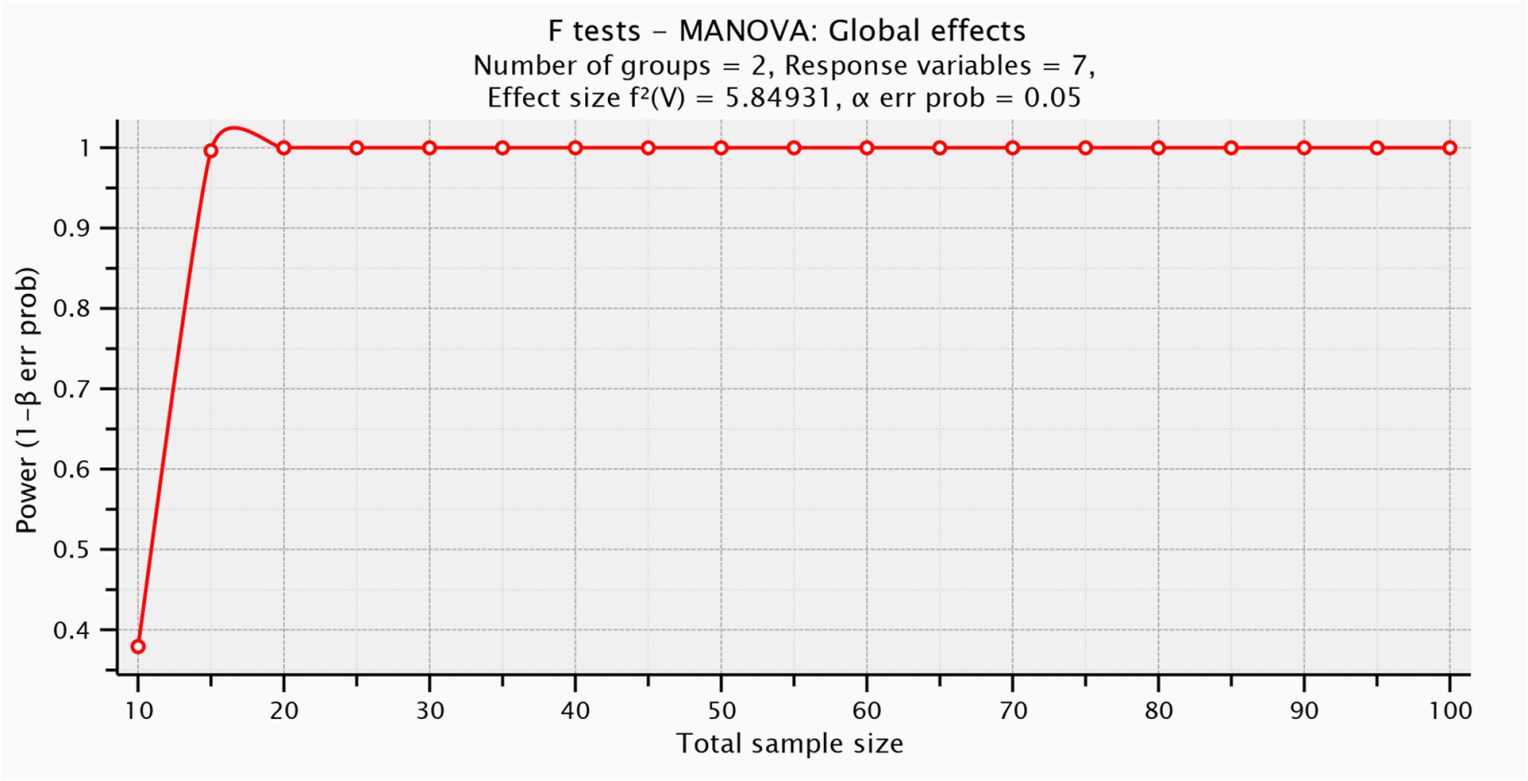
Plot of *post hoc* power analysis.

The number of participants included was limited by their availability during the designated data collection period. So, G*POWER statistical programming (version 3.1.9.2; Franz Faul, Universitat Kiel, Germany) was used to test *post hoc* power analysis [F tests- MANOVA: Global effects, *α* = 0.05, *β* = 0.5, number of predictors = 1, number of dependents = 7, Pillai V = 0.854, and sample size = 25] and revealed that the *post hoc* power analysis for this study was = 0.997] (Figure 1).

The statistical analysis was done utilizing Windows SPSS version 23 (SPSS, Inc., Chicago, IL). Participants’ characteristics and training profile of athletic females were compared between groups using an unpaired *t*-test. One-way between-subject MANOVA was used for comparing the iron status parameters between both tested groups. The extreme scores, variance homogeneity, as well as normality of the data were all analyzed. The difference analysis's parameter calculations necessitated this scan; hence it was carried out as a first step. Dot plots were generated for the hematological and serum parameters to visualize the distribution of the data across the groups. The *p*-value has been adjusted to <0.05.

## 3 Results

### 3.1 Demographic data

Both aerobic and anaerobic groups showed insignificant differences concerning age, weight, height, as well as BMI (*p* > 0.05) (Table 1).

**Table 1:** Demographic data of athletic females in both groups.

	Aerobic group (*n* = 13)	Anaerobic group (*n* = 12)	*p*-value
Age (years)	17.46 ± 1.27	17.58 ± 1.16	0.821^NS^
Weight (Kg)	53.31 ± 5.34	56.17 ± 5.57	0.677^NS^
Height (m)	1.62 ± 0.04	1.66 ± 0.06	0.073^NS^
BMI (Kg/m^2^)	20.34 ± 1.98	20.51 ± 2.57	0.389^NS^

Values are expressed as mean ± SD.

*p*, probability.

^NS^ *p* > 0.05 = non-significant.

### 3.2 Training profile

Regarding the training profile of athletic females, the results showed non-significant differences between both aerobic and anaerobic groups in age of participation in sport (MD 0.60; 95% CI −0.93: 2.12; *p* = 0.426), training experience (MD −0.56; 95% CI −1.69: 0.57; *p* = 0.319), and weekly training workload (MD −0.35; 95% CI −3.63: 2.94; *p* = 0.830) (Table 2).

**Table 2:** Training profile of athletic females in both groups.

	Aerobic group (*n* = 13)	Anaerobic group (*n* = 12)	MD (95% CI)	*p*-value
Age of participation in sport (years)	10.85 ± 2.23	10.25 ± 1.3	0.60 (−0.93: 2.12)	0.426^NS^
Training experience (years)	6.69 ± 1.44	7.25 ± 1.29	−0.56 (−1.69: 0.57)	0.319^NS^
Weekly training workload (hours)	14.15 ± 4.18	14.50 ± 3.73	−0.35 (−3.63: 2.94)	0.830^NS^

Values are expressed as mean ± SD.

*p*, probability.

^NS^ *p* > 0.05 = non-significant.

### 3.3 Hematological factors and iron status parameters

**Figure 2:**
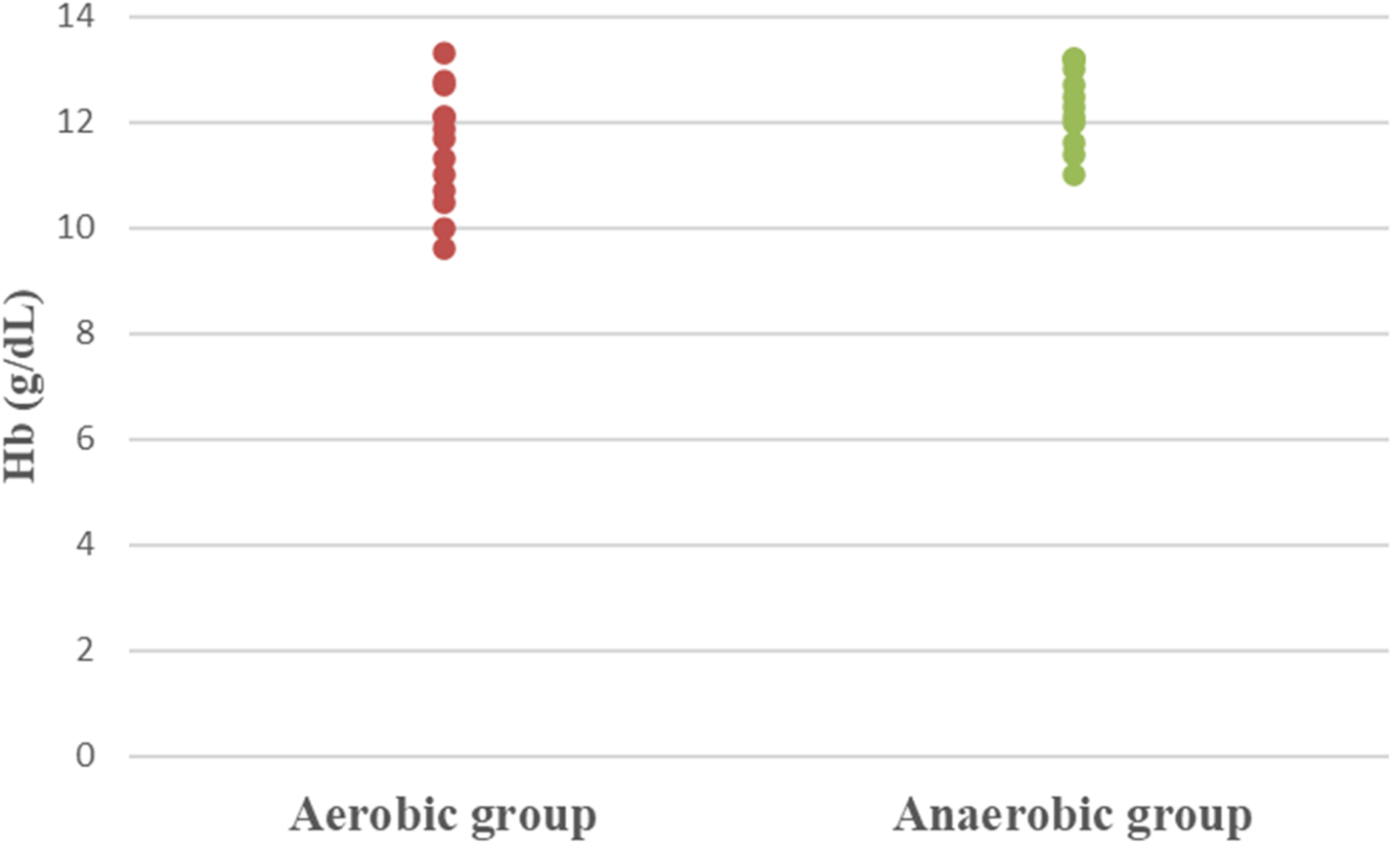
Dot plot of hemoglobin (Hb) for both groups.

**Figure 3:**
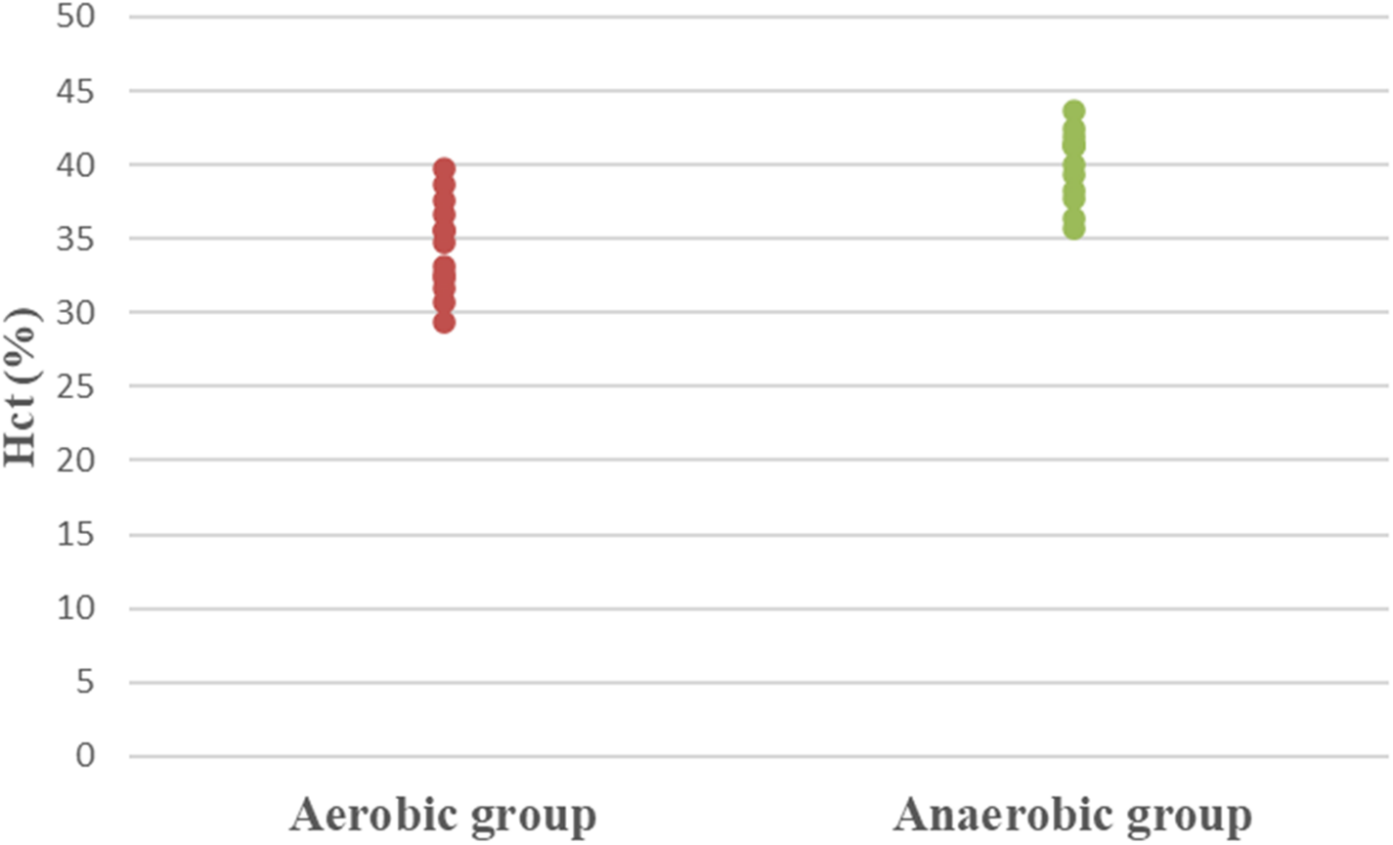
Dot plot of hematocrit (Hct) for both groups.

**Figure 4:**
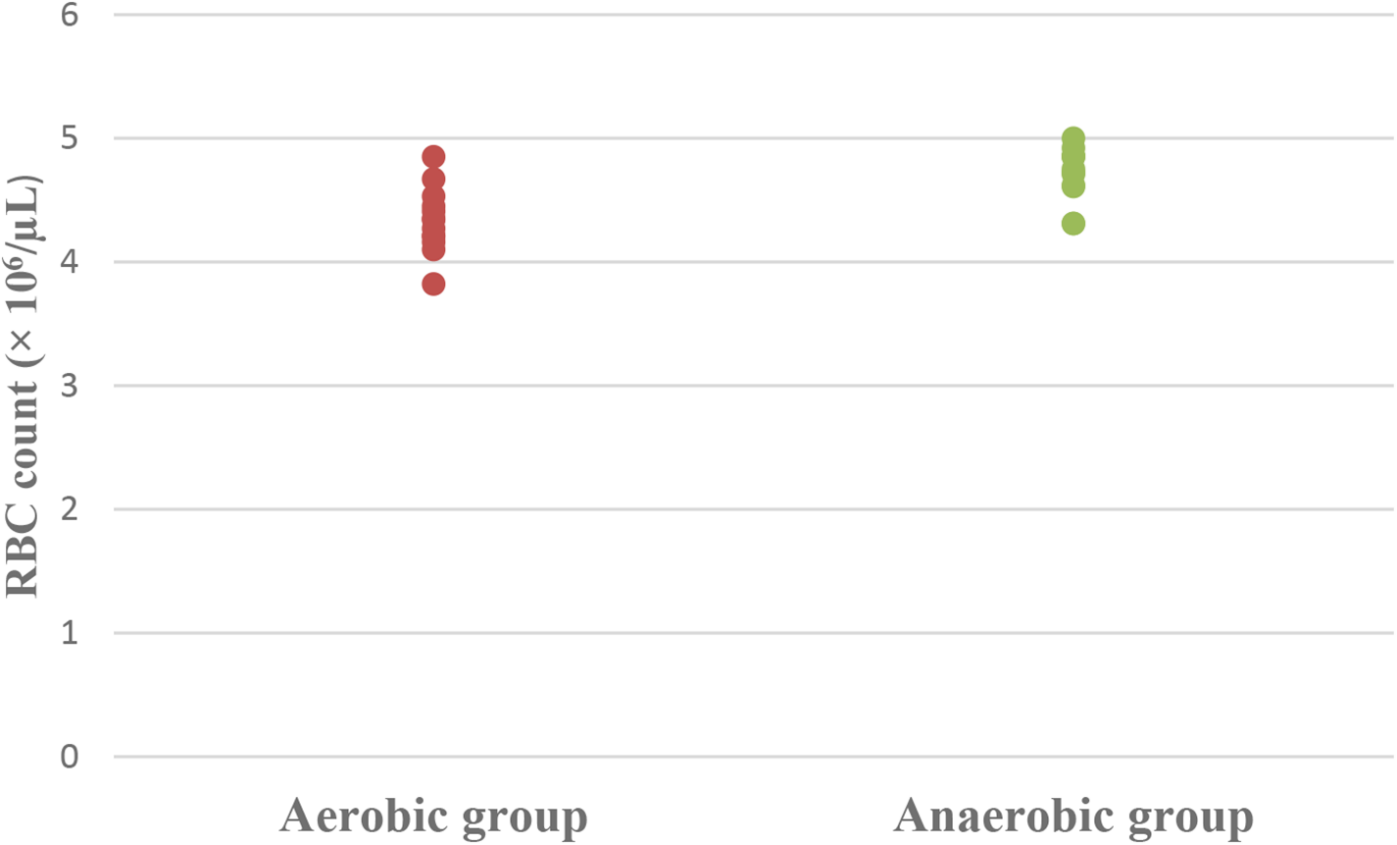
Dot plot of red blood cell (RBC) count for both groups.

**Figure 5:**
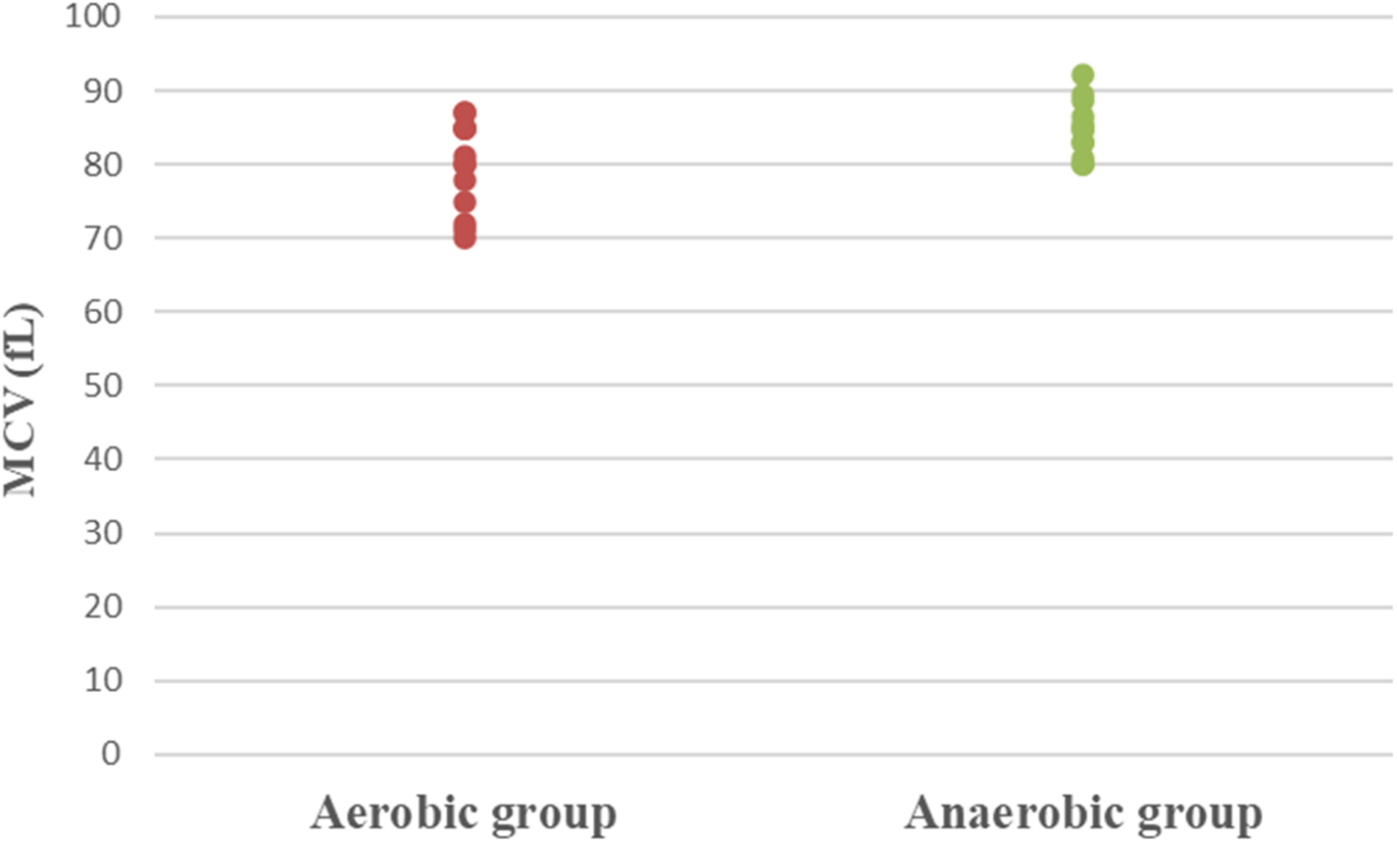
Dot plot of mean corpuscular volume (MCV) for both groups.

**Figure 6:**
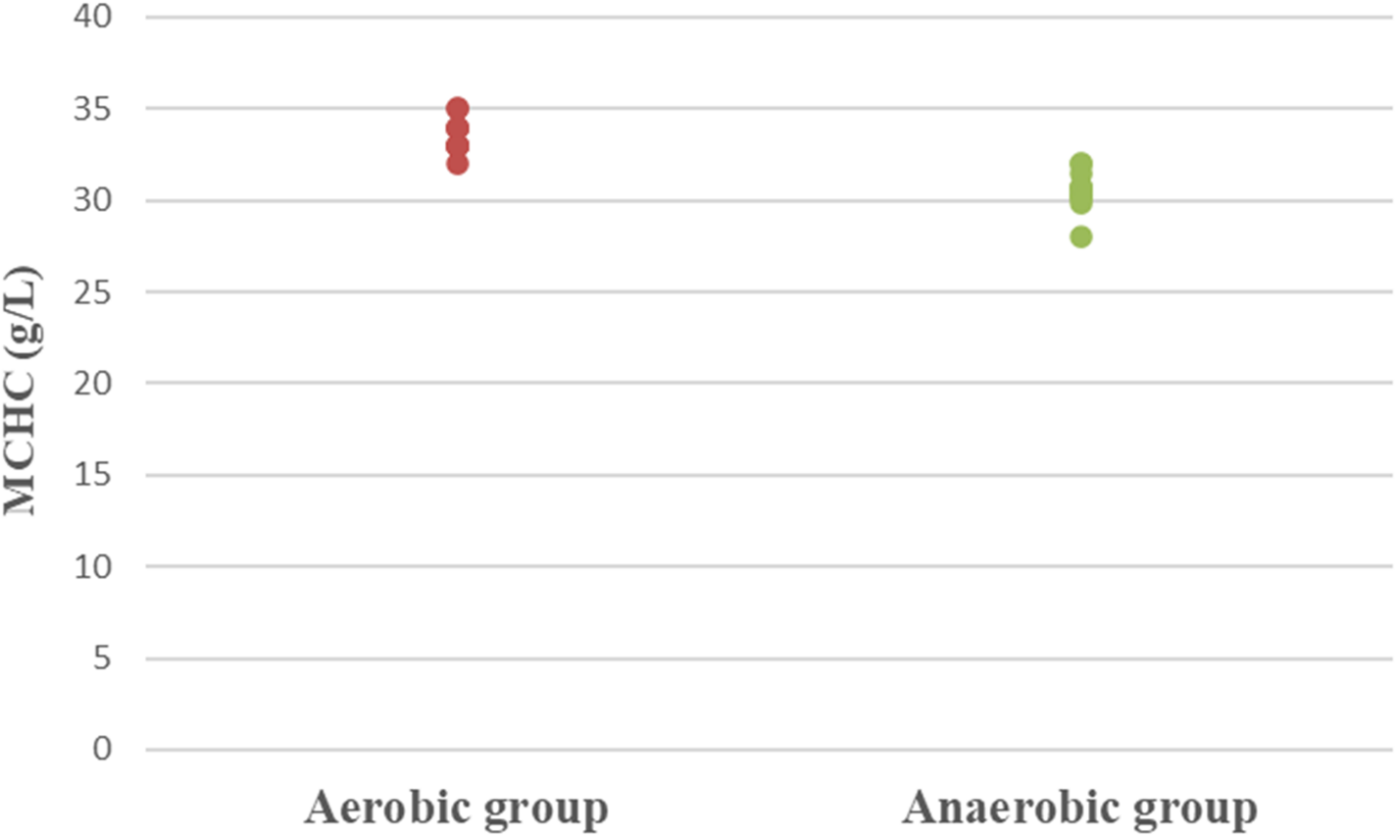
Dot plot of mean corpuscular hemoglobin concentration (MCHC) for both groups.

**Figure 7:**
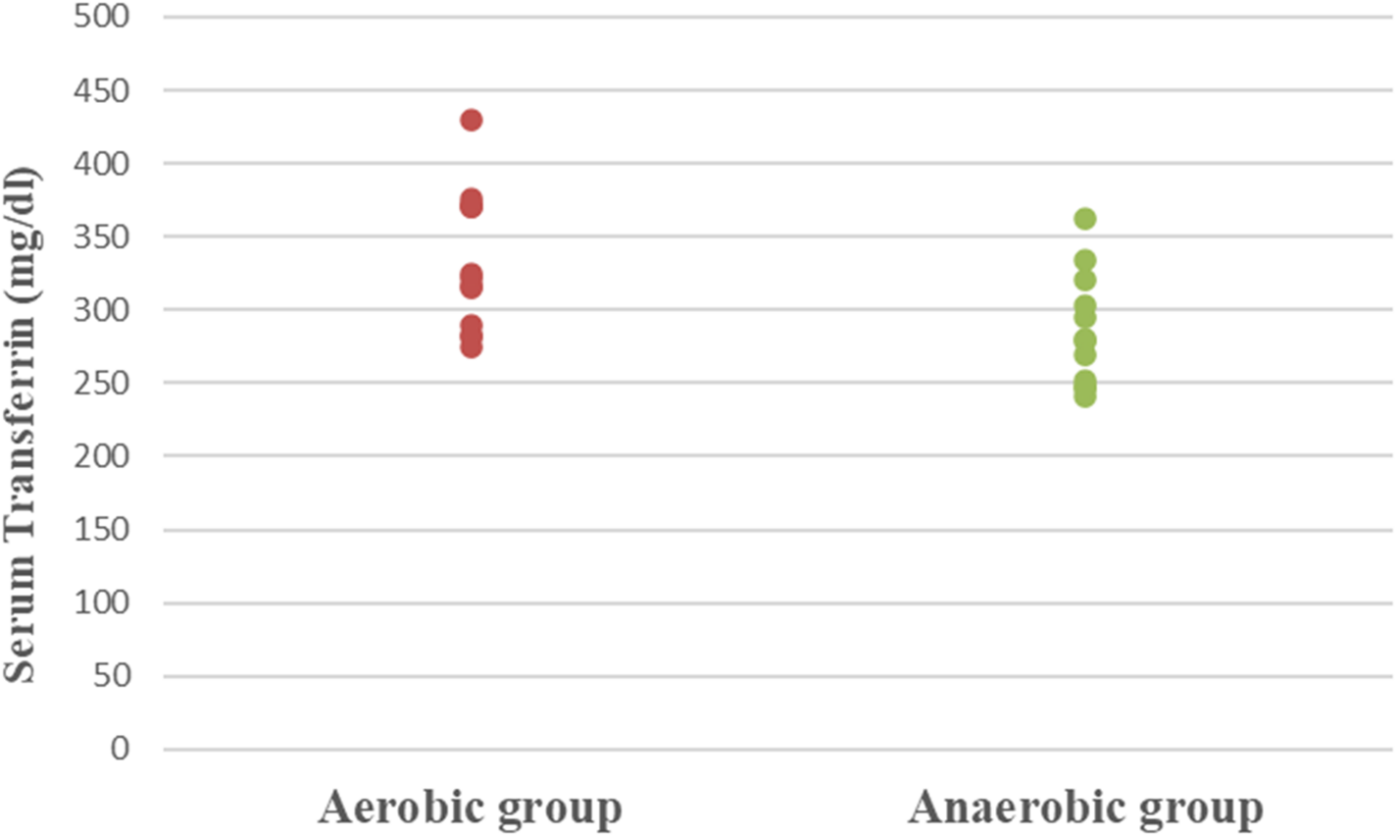
Dot plot of serum transferrin for both groups.

**Figure 8:**
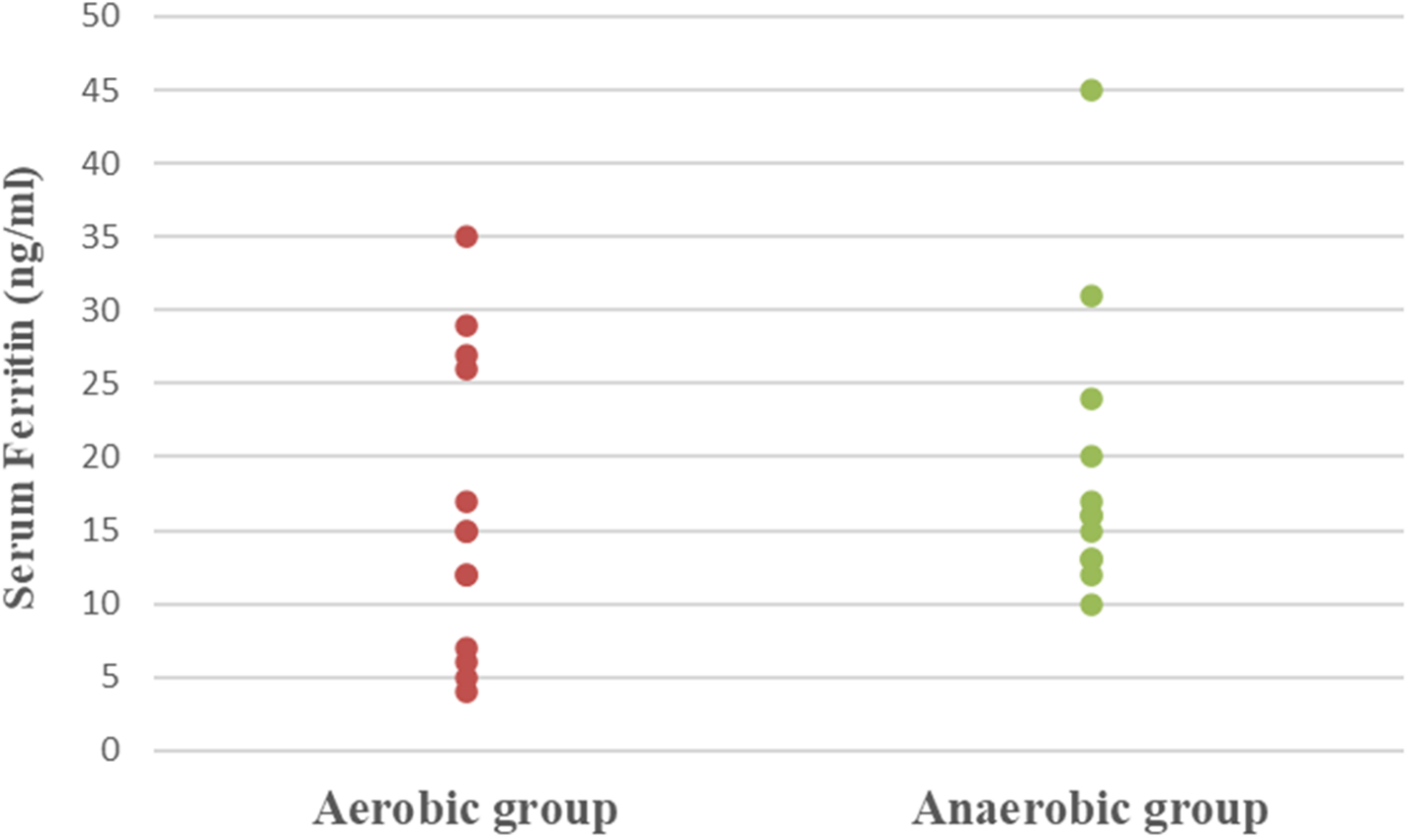
Dot plot of serum ferritin for both groups.

Concerning hematological factors and iron status parameters, the aerobic group had significantly lesser Hb (MD −0.84; 95% CI −1.63: −0.04; *p* = 0.041), Hct (MD −5.49; 95% CI −7.86: −3.12; *p* = 0.0001), RBC count (MD −0.37; 95% CI −0.57: −0.17; *p* = 0.001), and MCV (MD −5.15; 95% CI −9.41: −0.89; *p* = 0.020), as well as significantly higher MCHC (MD 2.99; 95% CI 2.18: 3.79; *p* = 0.0001) and serum transferrin (MD 46.77; 95% CI 10.95: 82.59; *p* = 0.013) than the anaerobic group. However, there was an insignificant difference in serum ferritin (MD −3.18; 95% CI −11.49: 5.13; *p* = 0.437) between both groups (Table 3; Figures 2–8).

**Table 3:** Hematological factors and iron status parameters for both groups.

	Aerobic group (*n* = 13)	Anaerobic group (*n* = 12)	MD (95% CI)	*p*-value
Hb (g/dl)	11.51 ± 1.12	12.35 ± 0.75	−0.84 (−1.63: −0.04)	0.041^S^
Hct (%)	34.45 ± 3.16	39.94 ± 2.49	−5.49 (−7.86: −3.12)	0.0001^S^
RBC count (×10^6^/µl)	4.34 ± 0.26	4.71 ± 0.22	−0.37 (−0.57: −0.17)	0.001^S^
MCV (fL)	79.69 ± 6.10	84.84 ± 3.83	−5.15 (−9.41: −0.89)	0.020^S^
MCHC (g/L)	33.54 ± 0.88	30.55 ± 1.07	2.99 (2.18: 3.79)	0.0001^S^
Serum transferrin (mg/dl)	332.77 ± 47.31	286.00 ± 38.33	46.77 (10.95: 82.59)	0.013^S^
Serum ferritin (ng/ml)	16.15 ± 10.13	19.33 ± 9.93	−3.18 (−11.49: 5.13)	0.437^NS^

Values are expressed as mean ± SD.

^S^ *p* < 0.01 = significant, *p*, probability.

^NS^ *p* > 0.05 = non-significant.

## 4 Discussion

Physical training induces iron status impairment in athletic females in the short term and over prolonged periods (36). Iron deficiency anemia refers to a condition where there is diminished production of red blood cells because of inadequate iron reserves within the body. It ranks as the leading nutritional disorder worldwide, contributing to about 50% of all cases of anemia (37). It remains a complex issue to diagnose and manage iron deficiency anemia effectively. Its diagnosis necessitates laboratory confirmation of anemia in addition to indications of depleted iron reserves (38). The iron storage protein, ferritin, is essential to maintain normal iron levels in the body. Iron is made available for essential cellular functions by ferritin, which also shields lipids, deoxyribonucleic acid, and proteins from iron's potentially harmful consequences (39).

The findings of the current study demonstrated significant reductions in Hb, Hct, RBC count, and MCV, as well as significant increases in MCHC and serum transferrin of athletic females in the aerobic group as compared with their counterparts in the anaerobic group.

### 4.1 Hematological factors (Hb, Hct, RBC count, MCV, and MCHC)

Regarding Hb, Hct, RBC count, and MCV, our findings agreed with Schumacher et al. (28), who reported reductions in levels of Hb, Hct, and RBC in endurance sports, mainly based on aerobic training, when compared with power sports, mainly based on anaerobic training (40). In contrast, Milic et al. (27) found non-significant differences between aerobic and anaerobic sports regarding Hb, Hct, and RBC, whereas there was a significant increase in MCV of the aerobic group. However, the disagreement between our findings and the earlier study's results could be attributed to differences in age, training profile, and sports disciplines of the studied sample.

The observed significant reductions in Hb, Hct, and RBC count, as well as MCV values in the aerobic group, coupled with a significant increase in MCHC, suggest potential specific training-induced adaptations. It is speculated that these adaptations may be influenced by both the type of exercise and its impact on the body's iron metabolism, though further investigation is required to confirm these findings.

The variability in the hematological factors between aerobic and anaerobic athletes may be explained by several modulating factors. Aerobic training is known to induce plasma volume expansion, a key adaptation aimed at improving oxygen delivery to muscles during prolonged exercise (28). This results in hemodilution, leading to lower Hb, Hct, and RBC counts in athletes engaged in endurance sports (41). Another possible explanation involves RBC destruction through exercise-induced hemolysis, which may be driven by several underlying mechanisms. The most recognized mechanism is foot strike hemolysis, resulting from the high stress generated on lower limb structures during long-distance running at foot strike, potentially inducing mechanical damage of RBC (42, 43). Another speculative mechanism contributing to exercise-elicited hemolysis is related to the increased oxidative stress associated with high oxygen flux, leading to increased vulnerability to oxidative damage and antioxidant depletion. Additionally, the osmotic imbalance, produced by the effects of exercise on altering the density and mean volume of RBCs, may predispose them to membrane damage and hemolysis. Moreover, it is possible that repeated contractile activity of exercising muscles during training induces compression and rupture of RBC. Similarly, internal organs’ vasoconstriction aiming to increase blood flow to exercising muscles may trigger RBC compression and hemolysis. Furthermore, metabolic alterations emerging during exercise training, such as hyperthermia and dehydration, are speculated to have triggering, accelerating, and amplifying effects on exercise-elicited hemolysis (44).

The significant increase in MCHC in the long-distance runners of the aerobic group aligned with the observations of Kratz et al. (45), who reported similar increases in MCHC among marathon runners both 4 h and 24 h post-race compared to pre-race levels. The increase in MCHC warrants careful interpretation, as it may provide insights into the physiological adaptations and potential risks associated with intense aerobic training. It is speculated that one potential explanation for the elevated MCHC in the aerobic group is the occurrence of acute intravascular hemolysis. Buys and Craven (46) suggested that increased MCHC levels could serve as an indicator of this phenomenon. Intravascular hemolysis in endurance athletes can result from various mechanisms, including mechanical stress, oxidative stress, and osmotic changes, but further research is needed to confirm this. The observed increase in MCHC could be a consequence of these hemolytic processes. As RBCs rupture, they release their Hb into the plasma, which may lead to a temporary increase in the concentration of Hb relative to the cell volume, resulting in higher MCHC values, but this is speculative.

### 4.2 Iron status (serum transferrin and ferritin)

Regarding transferrin, it is a key iron-binding protein in the blood, which plays a crucial role in iron transport and homeostasis (47). As reported by Ogun and Adeyinka (48), blood levels of transferrin are inversely related to body iron levels, with its production increasing in response to iron deficiency. This adaptive response aims to enhance iron uptake and transport efficiency when iron reserves are low. In the present study, the significantly elevated serum transferrin levels observed in the aerobic group are speculated to reflect the body's attempt to compensate for the higher prevalence of iron deficiency anemia in this group. This increase in transferrin production may be an effort to optimize iron absorption and maintain homeostasis in the face of iron deficiency.

Considering serum ferritin, it represents the principal mechanism for iron storage and is essential for maintaining iron homeostasis (49). In the current study, serum ferritin revealed an insignificant difference between both groups despite differences in other hematological factors, highlighting the complexity of iron homeostasis in response to different forms of physical activity. The lack of difference in ferritin levels, however, should be interpreted with caution. Schumacher et al. (50) reported that ferritin levels can be affected for several days following exercise due to an acute phase response. This physiological reaction can make accurate assessment of iron stores difficult or even impossible in the short term. In the current study, blood sampling occurred after only one day of rest, which may not have been sufficient for ferritin levels to stabilize. It is speculated that the ferritin levels in both groups might still have been reacting to recent physical exercise, potentially masking any true differences in iron storage between the aerobic and anaerobic training groups. This finding highlights the importance of considering the timing of blood sampling in relation to recent exercise when interpreting ferritin levels in athletes. Future studies should consider multiple time points for ferritin measurements.

### 4.3 Limitations and future research directions

The study has some limitations that warrant consideration. Firstly, the absence of a control group comprising non-athletic females could have provided a valuable reference for comparison, potentially enhancing the interpretation of the results. Secondly, focusing solely on adolescent females limits the direct applicability of the findings to other age groups or male athletes, emphasizing the necessity for broader demographic inclusion in future research. Finally, although the current study offers valuable insights into the hematological factors associated with different training modalities, it also highlights the complexity of assessing iron status in athletic females. Future research should explore a wider range of variables, including hormonal profiling, blood volume, and levels of hemolysis markers (e.g., lactate dehydrogenase enzyme and haptoglobin), to develop a more comprehensive understanding of iron status in the context of aerobic vs. anaerobic training among adolescent female athletes.

## 5 Conclusion

Aerobic training was associated with a worse impact on the hematological factors and iron status than anaerobic training in adolescent female athletes, except for ferritin levels, which showed no significant difference between aerobic and anaerobic training. Thus, athletic females participating in aerobic sports should be periodically evaluated, and educational programs should be designed during adolescence to improve their hematological factors and iron status. Education should focus on the importance of exercise modification as well as proper nutrition for optimizing health and sports performance.

## Data Availability

The raw data supporting the conclusions of this article will be made available by the authors, without undue reservation.
